# Parent–child correlation in energy and macronutrient intakes: A meta‐analysis and systematic review

**DOI:** 10.1002/fsn3.3957

**Published:** 2024-01-17

**Authors:** Farshad Teymoori, Mostafa Norouzzadeh, Hossein Farhadnejad, Mitra Kazemi Jahromi, Hamid Ahmadirad, Niloufar Saber, Mahdi Akbarzadeh, Maryam Zarkesh, Maryam S. Daneshpour, Parvin Mirmiran, Mohammadreza Vafa

**Affiliations:** ^1^ Nutrition and Endocrine Research Center, Research Institute for Endocrine Sciences Shahid Beheshti University of Medical Science Tehran Iran; ^2^ Department of Nutrition, School of Public Health Iran University of Medical Sciences Tehran Iran; ^3^ Endocrinology and Metabolism Research Center Hormozgan University of Medical Sciences Bandar Abbas Iran; ^4^ Cellular and Molecular Endocrine Research Center, Research Institute for Endocrine Sciences Shahid Beheshti University of Medical Sciences Tehran Iran

**Keywords:** child, dietary pattern, energy, macronutrient, meta‐analysis, parents

## Abstract

In the current study, we aimed to review the evidence from twin and family‐based studies that have assessed the familial similarity in intakes of energy and macronutrients among various parent–child pairs. The online literature databases, including Web of Science, PubMed, and Scopus, were searched up to December 2022 to find potentially eligible studies. We converted Pearson's, Spearman's, or intra‐class correlation coefficients to z's using Fisher's z transformation to obtain approximate normality and then calculated a mean and standard error (SE) of transformed correlation weighted by the sample sizes in the studies. We reported pooled *r* and 95% CI as our final results in five groups, including parent–child, mother–daughter, mother–son, father–daughter, and father–son. Twenty‐one eligible studies were included in this meta‐analysis, in which the sample size ranged from 33 and 4310. Our analysis showed that family resemblance in the intake of energy and macronutrients in various parent–offspring pairs was weak to moderate which could be different based on family pairs, nutrients, and studies. The highest similarity in dietary intakes was observed among the mother–daughter pair, which was for carbohydrate and protein intake, respectively. The lowest correlations in dietary intakes were found between mother–son or father–son pairs. Our meta‐analysis suggested that family similarity for intakes of energy and macronutrients was not strong in parent–child pairs. The highest correlation in dietary intake was mostly found in mother–daughter pairs. The weak similarities in dietary intake among parent–child pairs indicate the noticeable effect of the environment outside the family on individuals' dietary choices.

## Graphical abstract

Our meta‐analysis suggested that family similarity for intakes of energy and macronutrients was not strong in parent–child pairs. The highest correlation in dietary intake was mostly found in mother–daughter pairs. The weak similarities of dietary intake among parent–child pairs indicate the noticeable effect of the environment outside the family on individuals' dietary choices.

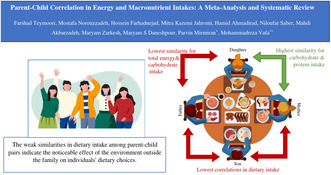

## 1 INTRODUCTION

Children's eating habits are influenced by both genetic and environmental factors, and parents can have an impact on both of these factors (Savage et al., 2007). They play a pivotal role as both gatekeepers and role models in influencing their children's nutrition beliefs and behaviors, which can have a significant impact on their offspring's dietary patterns and long‐term health outcomes (Beydoun & Wang, 2009). Hence, it is commonly believed that children's dietary intakes are strongly linked to their parents, owing to the combined influence of genetic and environmental factors on eating habits (Laskarzewski et al., 1980; Rossow & Rise, 1994). However, there is controversy regarding the results of studies on familial resemblance in dietary patterns. While some studies provide evidence supporting familial resemblance in dietary patterns (Laskarzewski et al., 1980; Oliveria et al., 1992; Pérusse et al., 1988; Rossow & Rise, 1994), other studies have found that the association is either very weak or non‐significant (Feunekes et al., 1997; Feunekes et al., 1998; Lahmann et al., 2017). This is likely because of the multifactorial nature of people's dietary patterns, with the family being responsible for only a part of it (Popkin, 2006). For example, as children age, their independence in food choices increases, and during this time, the influence of their peers on food choices becomes more apparent (Bogl et al., 2020; Nicklas et al., 2004). Thus, changes in children's dietary intake over time can also have an impact on the parent–child dietary association.

Considering these factors, it is important to conduct a systematic examination and quantification of the association between parent–child dietary intakes. Therefore, the main objective of this study is to conduct a systematic review and pairwise meta‐analysis of studies published since 1980 and evaluate the extent of correlation and similarity between parent–child dietary intakes. In addition, we performed meta‐regression analysis to compare correlations based on their type of parent–child relationship, dietary assessment approach, sample size, and other potential sources of heterogeneity. The results of this study are expected to contribute to our knowledge of the factors that influence dietary patterns among young people, which is important for developing effective public health interventions and policies aimed at promoting healthy eating habits and reducing the risk of chronic diseases.

## 2 METHOD

### 2.1 Systematic search

This systematic review and meta‐analysis was performed according to the preferred reporting items for systematic reviews and meta‐analyses (PRISMA) guidelines (Figure S1). Three major databases including PubMed, Scopus, and Web of Science were searched up to December 2022. Also, a manual search was performed in the Google Scholar database and reference list of found papers to find potential eligible studies. Two reviewers independently screened studies first by title and abstract and at the next level by full‐text reviewing.

### 2.2 Eligibility criteria

Each observational study that reports a correlation between energy and macronutrient intake among family member pairs is included in our study. There was no limitation for the age of participants and publication date. Familial relationships that were required for our study were: parent–child (PC), mother–child (MCH), father–child (FCH), father–son (FS), father–daughter (FD), mother–son (MS), and mother–daughter (MD).

Nevertheless, studies that weren't in English, didn't report sample size, reported *r* after implementing an intervention, or were conducted on unhealthy populations were excluded. In a few studies that did not announce the sample size for each group in the final analysis, but mentioned values were available at baseline, we calculated the approximate final sample size (e.g., we summed the parent's population with the child population to obtain parent–child sample size).

### 2.3 Data extraction

To have the minimum number of missing papers, two reviewers independently extracted the required data, shared their results, and resolved any inconsistencies by review. First author name, year of publication, region of studies, type of relationships, age of participants, the correlation coefficient presented by *r* (even intraclass, Pearson, or Spearman rank correlation coefficients) and its related sample size, dietary assessment method (either FFQ, dietary records or recalls) and unit of measurement was extracted from included studies.

### 2.4 Quality assessment

Newcastle‐Ottawa quality assessment scale for cross‐sectional studies was used to evaluate the studies' quality (Table S1). It measures studies' quality on 3 levels and checks them for selecting a representative sample, outcome and exposure assessment, comparability and cofounders' control, and using a proper statistical test. Eventually, a score of ≥7 out of 10 indicates the good quality of the studies.

### 2.5 Statistical analysis

The familial correlations of dietary intake of energy, carbohydrate, protein, and fat were evaluated during this study, and pooled *r* and 95% CI were reported for each of them in the following groups: (1) father–son, (2) father–daughter, (3) mother–son, (4) mother–daughter and (5) all (including mentioned groups besides to parent–child, father–child, and mother–child).

We converted Pearson's, Spearman's, or intra‐class correlation coefficients to z's using Fisher's z transformation to obtain approximate normality and then calculated a mean and standard error (SE) of transformed correlation weighted by the sample sizes in the studies (Donner & Rosner, 1980). Due to the number of studies and their conceptual heterogeneities, we run the random effect method for every analysis. *I* ^2^ statistic was used to present this heterogeneity and *p* heterogeneity <.05 was considered significant (Wang et al., 2011). As different studies report different units of nutrients for their measurements (e.g., calories, joules, gram, serving, percent of energy, or proportional terms like g/kg), in this case, we analyzed them in three levels. First, we pooled all correlation coefficients from every unit. If a study reports both gram and percent correlation coefficients for one variable, we analyzed them at the same time point. In the second and third levels, we analyzed studies that report the percent of energy and gram of intake, respectively.

Evaluation of publication bias was conducted using Egger's test, Begg's test, and visual inception of funnel plots. Regarding the number of included studies and to simplify our final results, Egger's test values were considered for reporting.

Regarding analyzing different nutrients in different pairs, and discrepancies in studies' population characteristics, there was conceptual and objective heterogeneity of reported correlations; so, we performed an unadjusted model of meta‐regression analysis to find some variables that could be a potential source of heterogeneity. In this order, we used Z and SE_Z_ statistics and reported the beta coefficients, 95% CI, *p*‐value, and tau^2^ (*τ* ^2^) for each variable. *τ* ^2^ statistic indicates between‐study variance and is an estimate of heterogeneity in the results. Therefore, if *τ* ^2^ is reduced by adjusting the effect of a variable, then the heterogeneity is reduced and that variable can be considered as a possible source of heterogeneity.

Therefore, meta‐regression was done based on the year of publication (published paper after 2000 vs. before 2000), children relationships (parent's correlation with girls vs. boys), parent relationships (children correlation with mothers vs. fathers), coexistence (parent–child correlations that not living together vs. living together), region (Asia, Oceania, and Africa vs. Europe and America), dietary assessment method (dietary records or recalls vs. FFQ), child's age (older than 18 years old vs. younger), sample size (larger than 500 people vs. lower than 500 people), and variable unit (percent of energy vs. gram of intake). Decision on studies that report age range was based on the population of different age groups or total mean age calculation using each group's mean age and its sample size.

Finally, we tested differences between the mean correlation of energy and macronutrient intake for each familial pair (FS, FD, MS, MG) using analysis of variance (ANOVA) and pairwise t‐test to find nutrients that significantly had different correlations in a family pair. We also examined differences among familial pairs for each nutrient correlation using ANOVA and pairwise *t*‐test to find family pairs who had the highest or lowest correlation coefficient of each nutrient. For all analyses, the significant levels were considered as *p* < .05. Statistical analysis was done using MedCalc software (version 20.218, Ostend, Belgium; https://www.medcalc.org; 2023) and STATA software version 17.0.

## 3 RESULTS

### 3.1 The literature searches

**FIGURE 1:**
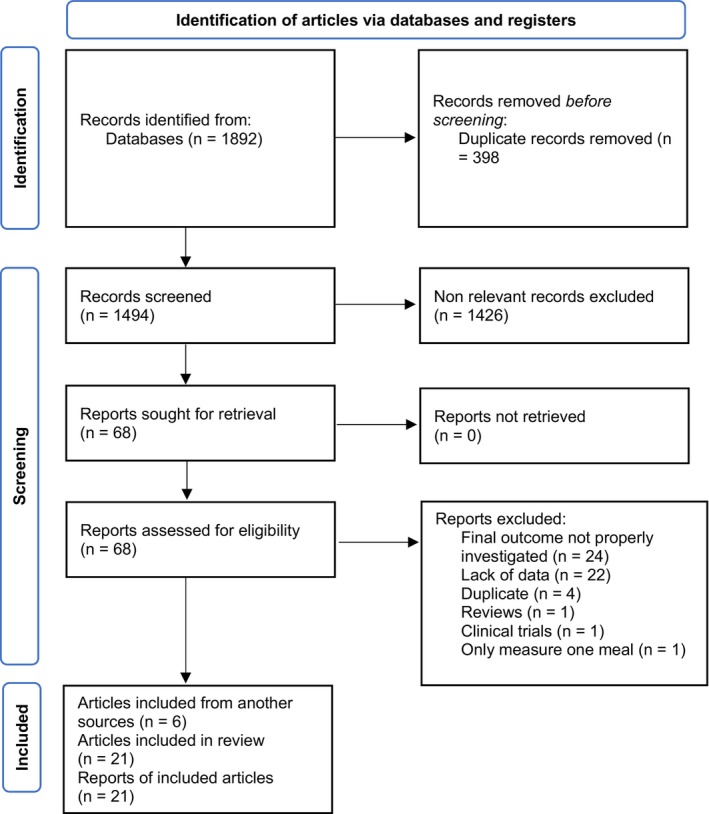
Flow diagram of selection of the published studies.

1892 publications were initially identified from databases. After excluding 398 duplicates and 1426, non‐relevant papers, 68 full‐text papers of potentially relevant studies were detected. Of 68 articles, 53 articles were eliminated based on the inclusion and exclusion criteria and 15 papers remained. In addition, 6 articles were obtained from other sources, and finally, 21 articles were included in this meta‐analysis (Figure 1).

### 3.2 Characteristics of included studies

Table 1 shows the data on the general characteristics of the 21 included articles (Adelekan & Adeodu, 1997; Beydoun & Wang, 2009; Bogl et al., 2017; Feunekes et al., 1997; Feunekes et al., 1998; Hosseini‐Esfahani et al., 2022; Lahmann et al., 2017; Laskarzewski et al., 1980; Lee & Park, 2015; Longbottom et al., 2002; Mitchell et al., 2003; Oliveria et al., 1992; Park et al., 2004; Pérusse et al., 1988; Rossow & Rise, 1994; Shrivastava et al., 2013; Stafleu et al., 1994; Stanton et al., 2003; Vauthier et al., 1996; Wang et al., 2009; Wroten et al., 2012). These studies were conducted in the USA (*n* = 7) (Beydoun & Wang, 2009; Laskarzewski et al., 1980; Mitchell et al., 2003; Oliveria et al., 1992; Stanton et al., 2003; Wang et al., 2009; Wroten et al., 2012), Netherlands (*n* = 3) (Feunekes et al., 1997; Feunekes et al., 1998; Stafleu et al., 1994), Korea (*n* = 2) (Lee & Park, 2015; Park et al., 2004), UK (*n* = 1) (Longbottom et al., 2002), Ireland (*n* = 1) (Shrivastava et al., 2013), Nigeria (*n* = 1) (Adelekan & Adeodu, 1997), Iran (*n* = 1) (Hosseini‐Esfahani et al., 2022), Australia (*n* = 1) (Lahmann et al., 2017), Norway (*n* = 1) (Rossow & Rise, 1994), Canada (*n* = 1) (Pérusse et al., 1988), Finland (*n* = 1) (Bogl et al., 2017), and France (*n* = 1) (Vauthier et al., 1996), and published in 1980 to 2022. The sample size in qualified studies evaluating the dietary resemblance among family members ranges from 33 to 4310. All included articles were high quality based on the NOS scores (Table S1). The correlation of macronutrients (in grams of intake or percent of energy or Serving/1000 Kcal) between 5 paired groups including PC, FS, FD, MS, and MD is reported in Table 1.

**TABLE 1:** The main characteristics of included studies assessed familial resemblance of macronutrient intake among family members.

Author, year, (quality score^a^)	Country	SS	Child age (year)	Dietary assessment	PC	FS	FD	MS	MD
Laskarzewski et al., 1980 (8/10)	USA	PC: 294	6–19	24‐h dietary recalls	Energy: (0.25) G: (CHO: 0.22)	‐	‐	‐	‐
Pérusse et al., 1988 (9/10)	Canada	PC: 1212	14.8	3‐day dietary record	Energy: (0.25) G: (CHO: 0.26, Pro: 0.26, Fat: 0.27) P: (CHO: 0.29, Pro: 0.27, Fat: 0.31)	‐	‐	‐	‐
Oliveria et al., 1992 (7/10)	USA	PC: 83 FS: 50 FD: 33 MS: 54 MD: 33	3–5	3‐day dietary records	Energy: (0.2) G: (CHO: 0.31, Pro: 0.37, Fat: 0.32)	Energy: (0.19) G: (CHO: 0.25, Pro: 0.25, Fat: 0.15)	Energy: (−0.03) G: (CHO: 0.03, Pro: 0.25, Fat: 0.12)	Energy: (0.03) G: (CHO: 0.28, Pro: 0.14, Fat: 0.4)	Energy: (0.22) G: (CHO: 0.47, Pro: 0.42, Fat: 0.49)
Stafleu et al., 1994 (9/10)	Netherlands	MD: 97 MGM: 97	25	FFQ (104 items)	Energy: (0.2)	‐	‐	‐	Energy: (MD: 0.22, MGM: 0.08) G: (Fat_MD_: 0.19, Fat_MGM_: 0.02)
Rossow & Rise, 1994 (8/10)	Norway	FCH: 544 MCH: 544	10–16	Questionnaire	G: (Fat_FCH_: 0.47, Fat_MCH_: 0.42)	‐	‐	‐	‐
Vauthier et al., 1996 (9/10)	France	PC: 1548 FS: 365 FD: 409 MS: 365 MD:409	14.2	3‐day food consumption diaries	Energy: (0.3) P: (CHO: 0.31, Pro: 0.33, Fat: 0.34)	Energy: (0.35) P: (CHO: 0.37, Pro: 0.36, Fat:0.39)	Energy: (0.33) P: (CHO: 0.26, Pro: 0.36, Fat: 0.28)	Energy: (0.24) P: (CHO: 0.28, Pro: 0.26, Fat: 0.31)	Energy: (0.26) P: (CHO: 0.35, Pro: 0.31, Fat: 0.4)
Adelekan & Adeodu, 1997 (9/10)	Nigeria	PC: 216	3.6	Three 24‐h dietary recalls	Energy: (0.39)	‐	‐	‐	‐
Feunekes et al., 1997 (7/10)	Netherlands	FS: 914 FD: 900 MS: 1003 MD: 998	1–30	2‐day diet record	‐	Energy: (0.19) G: (Fat: 0.28) P: (Fat: 0.4)	Energy: (0.24) G: (Fat: 0.35) P: (Fat: 0.39)	Energy: (0.09) G: (Fat: 0.2) P: (0.37)	Energy: (0.26) G: (Fat: 0.33) P: (Fat: 0.44)
Feunekes et al., 1998 (9/10)	Netherlands	FCH: 257 MCH: 296	15	Self‐administered FFQ	Energy: (FCH: 0.13, MCH: 0.19) P: (Fat_FCH_: 0.18, Fat_MCH_: 0.19)	‐	‐	‐	‐
Longbottom et al., 2002 (9/10)	UK	PC: 72	6.5	4‐day weighed food records	G: (Fat: 0.24)	‐	‐	‐	‐
Mitchell et al., 2003 (8/10)	USA	PC: 1038	>16	FFQ (102 items)	Energy: (0.14) G: (CHO: 0.05) P: (Pro: 0.1, Fat: 0.08)	‐	‐	‐	‐
Stanton et al., 2003 (8/10)	USA	MCH: 808 MS: 168 MD: 414	13	FFQ (35 items)	G: (Fat: 0.22)	‐	‐	G: (Fat: 0.11)	G: (Fat: 0.3)
Park, 2004 (9/10)^b^	Korea	FS: 96 FD: 83 MS: 98 MD: 83	13.3	FFQ	‐	Energy: (−0.03) P: (CHO: 0.12, Pro: 0.02, Fat: 0.2)	Energy: (0.2) P: (CHO: −0.1, Pro: 0.07, Fat: −0.01)	Energy: (0.1) P: (CHO: 0.23, Pro: 0.23, Fat: 0.19)	Energy: (0.27) P: (CHO: 0.17, Pro: 0.31, Fat: 0.09)
Wang et al., 2009 (9/10)	USA	MCH: 242 MS: 106 MD: 136	10–14	FFQ (152 Items)	Energy: (0.04) G: (Fat: 0.07) P: (Fat: 0.16)	‐	‐	Energy: (−0.24) G: (Fat: −0.21) P: (Fat: 0.19)	Energy: (0.26) G: (Fat: 0.3) P: (Fat: 0.11)
Beydoun & Wang, 2009 (9/10)	USA	PC: 4244 FS: 982 FD: 978 MS: 1156 MD: 1128	10.8	Two 24‐h dietary recalls	Energy: (0.22) G: (Fat: 0.24) P: (Fat: 0.01)	Energy: (0.29) G: (Fat: 0.27) P: (Fat: 0.01)	Energy: (0.14) G: (Fat: 0.18) P: (Fat: 0.02)	Energy: (0.23) G: (Fat: 0.28) P: (Fat: −0.04)	Energy: (0.26) G: (Fat: 0.24) P: (Fat: 0.02)
Shrivastava et al., 2013 (8/10)	Ireland	FCH: 898 MCH: 1125	5	FFQ (149 items)	Energy: (FCH: 0.13, MCH: 0.31) G: (CHO_FCH_: 0.15, CHO_MCH_: 0.27 Pro_FCH_: 0.13, Pro_MCH_: 0.27, Fat_FCH_: 0.05, Fat_MCH_: 0.25)	G: (CHO: 0.08, Pro: −0.05, Fat: −0.18)	G: (CHO: −0.08, Pro: −0.06, Fat: 0.05)	G: (CHO: −0.25, Pro: −0.18, Fat: −0.16)	G: (CHO: 0.06, Pro: 0.1, Fat: 0.07)
Wroten et al., 2012 (9/10)	USA	PC: 1300 FGF: 427 MGF: 1282 FGM: 494 MGM: 1402	4.4	24‐h dietary recalls	Energy: (0.48)	Energy: (FGF: −0.09)	Energy: (FGM: −0.24)	Energy: (MGF: −0.04)	Energy: (MGM: 0.06)
Lee & Park, 2015 (9/10)	Korea	PC: 4310 FS: 2122 FD: 2051 MS: 2259 MD: 2188	10.7	FFQ and 24‐h recall	‐	G: (Pro: −0.01)	G: (Pro: 0.01)	G: (Pro: −0.02)	G: (Pro: 0.01)
Bogl et al., 2017 (9/10)	Finland	PC: 1987 FS: 652 FD: 598 MS: 1410 MD: 1314	11	24‐h dietary recalls	Energy: (0.16) P: (CHO: 0.21, Pro: 0.26, Fat: 0.21)	Energy: (0.18) P: (CHO: 0.14, Pro: 0.23, Fat: 0.16)	Energy: (0.16) P: (CHO: 0.2, Pro: 0.28, Fat: 0.27)	Energy: (0.14) P: (CHO: 0.22, Pro: 0.26, Fat: 0.2)	Energy: (0.17) P: (CHO: 0.25, Pro: 0.25 Fat: 0.25)
Lahmann et al., 2017 (9/10)	Australia	MS: 1790 MD: 2244	20.6	FFQ (74 items)	‐	‐	‐	Energy: (0.01) G: (CHO: 0.21, Pro: 0.17, Fat: 0.15)	Energy: (0.15) G: (CHO: 0.21, Pro: 0.19, Fat: 0.24)
Hosseini‐Esfahani, 2022 (9/10)^c^	Iran	FS_1_: 1886 FD_1_: 1172 MS_1_: 1492 MD_1_: 1458 FS_2_: 520 FD_2_: 604 MS_2_: 706 MD_2_: 692	22	FFQ (168 items)	‐	Energy: (FS_1_: 0.07, FS_2_: 0.13) Serving/1000 Kcal (Pro_1_: 0.06, Pro_2_: 0.14)	Energy: (FD_1_: 0.07, FD_2_: 0.12) Serving/1000 Kcal (Pro_1_: 0.15, Pro_2_: 0.1)	Energy: (MS_1_: 0.14, MS_2_: −0.01) Serving/1000 Kcal (Pro_1_: 0.21, Pro_2_: 0.15)	Energy: (MD_1_: 0.22, MD_2_: 0.14) Serving/1000 Kcal (Pro_1_: 0.32, Pro_2_:0.08)

Abbreviations: FCH, father–child; FD, father–daughter; FFQ, food frequency questionnaire; FGF, father–grandfather; FGM, father–grandmother; FS, father–son; G, gram; MCH, mother–child; MD, mother–daughter; MGF, mother–grandfather; MGM, mother–grandmother; MS, mother–son; P, percent, CHO, carbohydrate; PC, parent–child; Pro, protein; SS, sample size.

^a^ Based on Newcastle‐Ottawa Scale (NOS).

^b^ Since this study did not announce the sample size by groups, the sample size for each group was obtained from the sum of the values mentioned at the beginning of the study.

^c^ The studied population is divided into two groups, including family members who live together (subgroup 1) and who live separately (subgroup 2).

The statistical distribution of qualified articles according to their characteristics is reported in Table 2. The included articles were categorized based on year of publication (before 2000 = 42.9% and 2000 and after =57.1%), region (Western countries = 76.2%, other = 23.8%), dietary assessment method (24‐h recalls or records = 47.6%, FFQ = 52.4%), child age (≤18 years = 81.0%, and >18 years = 19.0%), sample size (≤1000 = 42.9%, and >1000 = 57.1%), units of measurement (calorie = 18.0%, joules = 9.8%, gram = 33.5%, percent = 31.4%, serving = 1.0%, proportional = 5.7%, not reported = 0.5%), type of relationship (PC = 14.9%, FCH = 3.6%, MCH = 7.2%, FS = 16.0%, FD = 16.0%, MS = 20.1%, MD = 22.2%).

**TABLE 2:** Statistical distribution of included studies based on their characteristics in both absolute and proportional terms.

	21 included papers (percent)	194 analyzed correlations (percent)
Year of publication		
Before 2000	9 (42.9)	‐
2000 and after	12 (57.1)	‐
Region		
Western countries	16 (76.2)	‐
Other	5 (23.8)	‐
Dietary assessment method		
24‐h recalls or records	10 (47.6)	‐
FFQ	11 (52.4)	‐
Child age		
≤18 years	17 (81)	‐
>18 years	4 (19)	‐
Sample size		
≤1000	9 (42.9)	‐
>1000	12 (57.1)	‐
Units of measurement		
Calorie	‐	35 (18)
Joules	‐	19 (9.8)
Gram	‐	65 (33.5)
Percent	‐	61 (31.4)
Serving	‐	2 (1)
Proportional^a^	‐	11 (5.7)
Not reported	‐	1 (0.5)
Type of relationship		
PC	‐	29 (14.9)
FCH	‐	7 (3.6)
MCH	‐	14 (7.2)
FS	‐	31 (16)
FD	‐	31 (16)
MS	‐	39 (20.1)
MD	‐	43 (22.2)

Abbreviations: FCH, father–child; FD, father–daughter; FFQ, food frequency questionnaire; FGF, father–grandfather; FGM, father–grandmother; FS, father–son; MCH, mother–child; MD, mother–daughter; MGF, mother–grandfather; MGM, mother–grandmother; MS, mother–son; PC, parent–Child.

^a^ This category refers to the cases in which the unit is reported in a relative form (e.g., gram/body weight or serving/1000 kcal).

Table 3 indicates the pooled results of the meta‐analysis on macronutrient correlation in family members. Also, the range of correlations for macronutrient intake, pooled *r* (95% CI), the percent of heterogeneity, and the *p*‐value of the Egger test for evaluating the publication bias are stated in Table 3. The highest pooled *r* was reported for fat (percent of energy intake) (0.23), total CHO (percent of energy intake) (0.22), protein (percent of energy intake) (0.22) among FS, protein (percent of energy intake) (0.27), fat (percent of energy intake) (0.20), simple CHO (percent of energy, grams of intake, and servings/day) (0.19) among FD, protein (percent of energy intake) (0.25), total CHO (percent of energy intake) (0.23), and total CHO (percent of energy, grams of intake, and servings/day) (0.22) among MS, and total CHO (percent of energy intake) (0.27), protein (percent of energy intake) (0.26), and fat (percent of energy, grams of intake, and servings/day) (0.23) among MD. The highest pooled *r* among all members of the family was reported for protein (percent of energy intake) (0.25), total CHO (percent of energy intake) (0.24), and fat (percent of energy intake) (0.21).

**TABLE 3:** Pooled results of correlations of macronutrient intake among family members.

	All	FS	FD	MS	MD
Energy (*n*)^a^	17/56	8/9	8/9	10/11	11/13
Range	−0.09, 0.48	‐0.09, 0.35	−0.04, 0.33	−0.24, 0.24	0.06, 0.27
*Pooled r* (95% CI)^b^	0.16 (0.12, 0.19)	0.14 (0.06, 0.23)	0.14 (0.05, 0.22)	0.05 (−0.02, 0.13)	0.19 (0.14, 0.23)
Heterogeneity (%)^c^	91.66	89.25	88.95	92.01	74.72
Eager (*p*‐value)^d^	.51	.71	.45	.45	.48
Total CHO^e^ (*n*)^a^	9/31	5/5	5/6	5/5	6/6
Range	−0.25, 0.47	0.08, 0.31	−0.25, 0.26	0.21, 0.28	0.06, 0.47
*Pooled r* (95% CI)^b^	0.18 (0.14, 0.23)	0.19 (0.06, 0.31)	0.01 (−0.16, 0.19)	0.22 (0.19, 0.25)	0.22 (0.13, 0.31)
Heterogeneity (%)^c^	91.56	81.41	94.70	**0.00**	89.08
Eager (*p*‐value)^d^	.91	.82	.82	.22	.53
Total CHO^f^ (*n*)^a^	4/15	3/3	3/3	3/3	3/3
Range	−0.10, 0.37	0.12, 0.37	−0.10, 0.26	0.22, 0.28	0.17, 0.35
*Pooled r* (95% CI)^b^	0.24 (0.20, 0.28)	0.22 (0.03, 0.38)	0.15 (0.01, 0.29)	0.23 (0.18, 0.27)	0.27 (0.19, 0.35)
Heterogeneity (%)^c^	70.86	86.88	77.72	0.0	57.21
Eager (*p*‐value)^d^	.48	.94	.35	.64	.96
Total CHO^g^ (*n*)^a^	5/15	‐	2/3	‐	3/3
Range	−0.25, 0.47	‐	−0.25, 0.03	‐	0.06, 0.47
*Pooled r* (95% CI)^b^	0.13 (0.05, 0.21)	‐	−0.13 (−0.28, 0.01)	‐	0.18 (0.03, 0.32)
Heterogeneity (%)^c^	93.86	‐	82.82	‐	91.65
Eager (*p*‐value)^d^	.88	‐	.92	‐	.85
Simple CHO^e^ (*n*)^a^	6/18e	2/3	2/3	3/4	4/6
Range	−0.02, 0.41	−0.02, 0.19	0.10, 0.41	−0.02, 0.16	0.10, 0.30
*Pooled r* (95% CI)^b^	0.16 (0.11, 0.20)	0.11 (−0.02, 0.24)	0.19 (0.05, 0.33)	0.10, (0.02, 0.17)	0.15 (0.10, 0.20)
Heterogeneity (%)^c^	86.36	85.77	87.55	79.79	**53.31**
Eager (*p*‐value)^d^	.89	.88	.31	.70	.39
Protein^e^ (*n*)^a^	10/43	7/8	7/8	8/9	8/9
Range	−0.18, 0.37	−0.05, 0.36	−0.06, 0.36	−0.18, 0.26	0.01, 0.42
*Pooled r* (95% CI)^b^	0.16 (0.12, 0.20)	0.12 (0.02, 0.21)	0.13 (0.03, 0.24)	0.13, (0.03, 0.22)	0.20 (0.11, 0.28)
Heterogeneity (%)^c^	94.44	90.75	93.24	94.52	93.86
Eager (*p*‐value)^d^	.10	.26	.25	.86	.42
Protein^f^ (*n*)^a^	5/16	3/3	3/3	3/3	3/3
Range	0.02, 0.36	0.02, 0.36	0.07, 0.36	0.23, 0.26	0.25, 0.31
*Pooled r* (95% CI)^b^	0.25 (0.22, 0.29)	0.22 (0.07, 0.37)	0.27 (0.15, 0.38)	0.25 (0.21, 0.30)	0.26 (0.22, 0.30)
Heterogeneity (%)^c^	74.33	81.35	70.11	**0.0**	**0.0**
Eager (*p*‐value)^d^	.69	.68	.50	.36	.44
Protein^g^ (*n*)^a^	5/19	3/3	3/3	4/4	4/4
Range	−0.18, 0.42	−0.05, 0.25	−0.06, 0.25	−0.18, 0.17	0.01, 0.42
*Pooled r* (95% CI)^b^	0.08 (0.03, 0.14)	0.0 (−0.08, 0.07)	−0.01 (−0.08, 0.06)	0.01 (−0.14, 0.17)	0.12 (0.01, 0.23)
Heterogeneity (%)^c^	93.83	**49.78**	67.06	95.30	92.57
Eager (*p*‐value)^d^	.34	.59	.70	.81	.72
Fat^e^ (*n*)^a^	16/64	7/9	7/9	10/13	11/15
Range	−0.21, 0.49	−0.18, 0.40	−0.01, 0.39	−0.21, 0.40	0.02, 0.49
*Pooled r* (95% CI)^b^	0.20 (0.16, 0.24)	0.19 (0.06, 0.31)	0.19 (0.08, 0.30)	0.15 (0.06, 0.24)	0.23 (0.16, 0.30)
Heterogeneity (%)^c^	94.10	95.35	94.11	93.97	92.51
Eager (*p*‐value)^d^	.55	.77	.92	.75	.83
Fat^f^ (*n*)^a^	9/30	5/5	5/5	6/6	6/6
Range	−0.04, 0.44	0.01, 0.40	−0.01, 0.39	−0.04, 0.37	0.02, 0.44
*Pooled r* (95% CI)^b^	0.21 (0.15, 0.26)	0.23 (0.05, 0.40)	0.20 (0.03, 0.36)	0.20 (0.05, 0.34)	0.23 (0.06, 0.38)
Heterogeneity (%)^c^	95.50	95.85	94.99	95.32	96.02
Eager (*p*‐value)^d^	.22	.79	.88	.79	.95
Fat^g^ (*n*)^a^	9/31	4/4	4/4	7/7	8/9
Range	−0.21, 0.49	−0.18, 0.28	0.05, 0.35	−0.21, 0.40	0.02, 0.49
Pooled *r* (95% CI)^b^	0.18 (0.13, 0.22)	0.13 (−0.08, 0.34)	0.18 (0.02, 0.34)	0.10 (−0.01, 0.22)	0.23 (0.15, 0.30)
Heterogeneity (%)^c^	91.06	95.99	94.29	93.31	86.56
Eager (*p*‐value)^d^	.35	.59	.89	.49	.73

Abbreviations: CHO, carbohydrate; FD, father–daughter; FS, father–son; MG, mother–daughter; MS, mother–son.

^a^ Presented as the number of papers/number of studies.

^b^ Fisher's transformed.

^c^ Not Significant heterogeneities are bolded.

^d^ Significant publication biases are bolded.

^e^ Percent of energy, grams of intake, and servings/day.

^f^ Percent of energy intake.

^g^ Grams of intake.

Regarding the diversity of included articles in essential variables, there is significant heterogeneity in the meta‐analysis on the correlation of macronutrient intake in family members except for total CHO (percent of energy, grams of intake, and servings/day) among MS, Simple CHO (percent of energy, grams of intake, and servings/day) among MD, protein (percent of energy intake) among MD and MS, protein (grams of intake) among FS. We evaluated the publication bias using the *p*‐value of the Egger test, no significant publication bias was observed in our meta‐analysis.

The meta‐regression on energy and macronutrient intakes to find the potential sources of heterogeneity including year (published paper after 2000 vs before 2000), children (parent's correlation with girls vs boys), parent (children correlation with mothers vs fathers), coexistence (parent–child correlations that not living together vs living together), region (Others including Asia, Oceania, and Africa vs Europe and America), dietary assessment (dietary intakes assessed by records or recalls vs FFQ), child age (children older than 18 years old vs younger.), sample size (study population larger than 500 people vs lower than 500 people), and variable unites (percent of energy vs gram of intake) among family members is shown in Table 4.

**TABLE 4:** Meta‐regression analysis for energy and macronutrient intake among all family members^a^.

Unadjusted model				
Variable	Coefficient	95% CI	*p*‐value	*τ* ^2^
Energy				.016
Year^b^	**−.09**	**−0.17, −0.02**	**.01**	.014
Children^c^	.07	−0.005, 0.15	.06	.014
Parent^d^	.01	−0.06, 0.10	.69	.018
Coexistence^e^	**−.18**	**−0.26, −0.09**	**.001**	.011
Region^f^	−.04	−0.13, 0.03	.26	.016
Dietary Assessment^f^	**.14**	**0.08, 0.21**	**.001**	.010
Child age^h^	**−.14**	**−0.21, −0.07**	**.001**	.011
Sample size^i^	.02	−0.05, 0.10	.51	.016
Fat				.022
Year^b^	**−.20**	**−0.26, −0.13**	**.001**	.012
Children^c^	.00	−0.10, 0.10	.96	.026
Parent^d^	.02	−0.07, 0.11	.63	.024
Coexistence^e^	**−.27**	**−0.40, −0.14**	**.001**	.017
Region^f^	−.06	−0.20, 0.08	.41	.022
Dietary Assessment^g^	**.11**	**0.03, 0.19**	**.004**	.020
Child age^h^	**−.18**	**−0.28, 0.07**	**.001**	.018
Sample size^i^	.05	−0.02, 0.13	.18	.22
Variable unites^j^	.01	−0.06, 0.09	.72	.022
CHO				.018
Year^b^	**−.17**	**−0.26, −0.07**	**.001**	.011
Children^c^	.00	−0.16, 0.15	.95	.024
Parent^d^	.06	−0.07, 0.20	.35	.022
Coexistence^e^	**−.27**	**−0.38, −0.17**	**.001**	.007
Region^f^	−.04	−0.18, 0.10	.56	.018
Dietary Assessment^g^	**.17**	**0.08, 0.26**	**.001**	.010
Child age^h^	**−.19**	**−0.29, 0.10**	**.001**	.010
Sample size^i^	.00	−0.11, 0.10	.93	.018
Variable unites^j^	**.10**	**0.00, 0.20**	**.04**	.014
Protein				.017
Year^b^	**−.18**	**−0.27, −0.10**	**.001**	.011
Children^c^	**−.13**	**−0.23, −0.03**	**.01**	.016
Parent^d^	.04	−0.05, 0.14	.35	.017
Coexistence^e^	**−.16**	**−0.26, −0.06**	**.002**	.013
Region^f^	**−.09**	**−0.18, −0.01**	**.02**	.015
Dietary Assessment^g^	**.19**	**0.13, 0.26**	**.001**	.008
Child age^h^	**−.10**	**−0.19, −0.01**	**.01**	.015
Sample size^i^	−.04	−0.14, 0.05	.33	.017
Variable unites^j^	**.13**	**0.05, 0.21**	**.002**	.012

*Note:* The bold values in the table are statistically significant.

^a^ All papers that report energy and macronutrient intake in all familial pairs were included.

^b^ Published paper after 2000 versus before 2000.

^c^ Parent's correlation with girls versus boys.

^d^ Children correlation with mothers versus fathers.

^e^ Parent–child correlations that not living together versus living together.

^f^ Others (Asia, Oceania, and Africa) versus Europe and America.

^g^ Dietary intakes assessed by records or recalls versus FFQ.

^h^ Children older than 18 years old versus younger.

^i^ Study population larger than 500 people versus lower than 500 people.

^j^ Units of variable (percent of energy versus gram of intake).

### 3.3 Meta‐regression for family members

There is an inverse relationship between year (coefficient: −.09; 95% CI: −0.17, −0.02; *p* = .010), coexistence (coefficient: −.18; 95% CI: −0.26, −0.09; *p* = .001), child age (coefficient: −.14; 95% CI: −0.21, −0.07; *p* = .001), and correlation of energy; however, dietary assessment (coefficient: .14; 95% CI: 0.08, 0.21; *p* = .001) had a positive relationship with energy correlation. The *τ* ^2^ value has been changed from .016 to .014, .011, .010, and .011 for the year, coexistence, dietary assessment, and child age, respectively.

Also, the fat correlation has an inverse relationship with year (coefficient: −.20; 95% CI: −0.26, −0.13; *p* = .001), coexistence (coefficient: −.27; 95% CI: −0.40, −0.14; *p* = .001), and child age (coefficient: −.18; 95% CI: −0.28, −0.07; *p* = .001); however, correlation of fat has a direct association with dietary assessment (coefficient: .11; 95% CI: 0.03, 0.19; *p* = .004). This meta‐regression indicated that the *τ* ^2^ value was reduced from .022 to .012, .017, .020, and .018 for the year, coexistence, dietary assessment, and child age, respectively.

The correlation of carbohydrates inversely was related to year (coefficient: −.17; 95% CI: −0.26, −0.07; *p* = .001), coexistence (coefficient: −.27; 95% CI: −0.38, −0.17; *p* = .001), child age (coefficient: −.19; 95% CI: −0.29, −0.10; *p* = .001), and variable unites (coefficient: .10; 95% CI: 0.00, 0.20; *p* = .040); however, carbohydrate correlation positively associated with dietary assessment (coefficient: .17; 95% CI: 0.08, 0.26; *p* = .001). The *τ* ^2^ value has been reduced from .018 to .011, .007, .010, .010, and .014 for the year, coexistence, dietary assessment, child age, and variable units, respectively.

The correlation of protein inversely related to year (coefficient: −.18; 95% CI: −0.27, −0.10; *p* = .001), children (coefficient: −.13; 95% CI: −0.23, −0.03; *p* = .010), coexistence (coefficient: −.16; 95% CI: −0.26, −0.06; *p* = .002), region (coefficient: −.09; 95% CI: −0.18, −0.01; *p* = .020), and child age (coefficient: −.10; 95% CI: −0.19, −0.01 *p* = .010); however, correlation of fat has a positive relationship with dietary assessment (coefficient: .19; 95% CI: 0.13, 0.26; *p* = .001) and variable unites (coefficient: .13; 95% CI: 0.05, 0.21; *p* = .002). This meta‐regression shows that the *τ* ^2^ value was reduced from .017 to .011, .016, .013, .015, .008, .015, and .012 for the year, children, coexistence, region, dietary assessment, child age, and variable units, respectively.

The difference in mean *r* between family members paired with energy and macronutrient intake is reported in Table 5. The findings indicated a significant difference in mean *r* between MS and MD in energy intake (*p*‐value <.05). However, there was no significant difference in the mean *r* among other family members paired for intakes of energy and macronutrients.

**TABLE 5:** Comparison of mean *r* differences between energy and macronutrient intake among family member pairs^a^.

Nutrients	Familial pairs correlations				Overall and pairwise *p*‐values						
	1‐FS	2‐FD	3‐MS	4‐MD	*p*‐ANOVA	*p*_1–2_	*p*_1–3_	*p*_1–4_	*p*_2–3_	*p*_2–4_	*p*_3–4_
Energy	0.14	0.13	0.04	0.19	**.04**	.86	.09	.31	.12	.23	**.005**
Carbohydrate	0.19	0.06	0.16	0.25	.30	.22	.76	.55	.32	.06	.34
Protein	0.12	0.14	0.13	0.22	.48	.78	.88	.17	.89	.27	.21
Fat	0.18	0.18	0.15	0.23	.65	.96	.64	.51	.67	.48	.21

Abbreviations: FD, father–daughter; FS, father–son; MG, mother–daughter; MS, mother–son.

^a^ Significance level considered *p*‐value <0.05 and significant differences are bolded.

Table 6 shows the mean *r* differences in family member pairs including FS, FD, MS, and MD according to energy and macronutrient intakes. There are no significant differences in paired groups consisting of energy and macronutrient intakes among all paired family members.

**TABLE 6:** Comparison of mean *r* differences between family member pairs based on their energy intake and macronutrients.

Familial pairs	Correlations				Overall and pairwise *p*‐values						
	1‐energy	2‐carbohydrate	3‐protein	4‐fat	*p*‐ANOVA	*p*_1–2_	*p*_1–3_	*p*_1–4_	*p*_2–3_	*p*_2–4_	*p*_3–4_
FS	0.14	0.19	0.12	0.18	.79	.56	.81	.54	.44	.95	.41
FD	0.13	0.06	0.14	0.18	.50	.38	.85	.44	.31	.13	.58
MS	0.04	0.16	0.13	0.15	.41	.19	.25	.13	.77	.92	.81
MD	0.19	0.25	0.22	0.23	.82	.39	.67	.46	.64	.75	.82

Abbreviations: FD, father–daughter; FS, father–son; MG, mother–daughter; MS, mother–son.

## 4 DISCUSSION

In this meta‐analysis study, we focused on the results of various studies that had investigated the possible family similarity in dietary intakes (energy and macronutrients) between various parent–child pairs including (parent–child, father–son, father–daughter, mother–son, and mother–daughter). The pooled *r* based on studies results showed that family correlation of dietary intakes between various parent–child pairs can be varied according to types of parent–child dyad, nutrients, and studies. The correlation reported for dietary intakes, including energy, carbohydrate, fat, and protein was weak to moderate based on different parent–child pairs. The highest family similarity in dietary intakes was found among mother–daughter pairs, which included carbohydrates and proteins, respectively. The lowest family correlations in energy and macronutrient intakes were mainly found between mother–son or father–son pairs. Various factors, including the year of publication of the paper, the dietary assessment approach, the coexistence of parent–child, the child gender, and child age were the sources of heterogeneity in the results of this meta‐analysis. The interesting results of this meta‐analysis provided insight into the extent of the parental effect on offspring food habits and dietary intakes and showed that there was a weak‐to‐moderate correlation in intakes of energy and macronutrients among various parent–child dyads. The weak to moderate family similarity in dietary intakes among parent–child pairs suggested that although dietary intakes of individuals may somewhat be influenced by genetic affinities between the parents and offspring, the noticeable effect of shared and non‐shared environmental factors should not be missed. Previous studies that reported weak correlations for dietary intakes between parent–child pairs suggested that dietary behaviors of offspring, especially in adolescence and youth periods, can be influenced by various environmental factors in addition to household factors, including the influence of friends and peers, the effect of food environments of community, workplace, and school, the influence of the type of environment and programs used to spend leisure time such as television viewing, as well as personal factors, such as self‐esteem, autonomy, and self‐image (Beydoun & Wang, 2009; Boynton‐Jarrett et al., 2003; French et al., 2001; Salvy et al., 2007; Satia et al., 2001; Shariff & Yasin, 2005).

This meta‐analysis of the findings of previous studies shows that family similarities in energy and macronutrient intake in mother–daughter pairs are somewhat stronger than in other parent–child pairs. Although the reasons for these findings are not fully known, some factors can justify this interesting finding (Hosseini‐Esfahani et al., 2022; Park et al., 2004; Wang et al., 2009); in most societies, mothers and daughters spend more time during the day than fathers and sons in the home environment and may have a greater joint role in the process of cooking and preparing food for the family's main meals and hence their food choices may be more similar in meals throughout a day (Hosseini‐Esfahani et al., 2022). However, sons may spend more time outside the home environment with peers and schoolmates, and under the influence of these people, they may have different food choices than their family and parents (Hosseini‐Esfahani et al., 2022; Park et al., 2004). So, this point has caused the correlation of dietary intake between parents–son, especially father–son, has been reported to be weak in previous studies. Also, boys and girls potentially have different biological, psychosocial, behavioral, and physiological differences, which can explain the difference in the effect of genetic and environmental interaction on family similarity reported for mother–daughter pairs compared to other parent–child pairs (Wang et al., 2009). Regarding that, based on biological differences, the period of physical growth, maturity, and the stage of body development of sons and daughters occurred at different times of life, therefore, the nutritional needs of sons and daughters may be differed according to the influence of these factors that subsequently cause differences in dietary behaviors between them during adolescence and the later stages of life. For instance, at a certain age during adolescence, girls have fully matured physically, but boys are still growing and maturing physically. Moreover, physiological differences in sons and daughters may affect differences in similarities of dietary intakes between parents and offspring; because of possible differences in body parts and physical strength, sons may be more physically active than daughters, which may cause them to have different food intake during the day. Generally, according to the points mentioned above, the effects of the home environment, outdoor environments (school, workplace, restaurant, etc.), parents, peers, classmates, and friends on sons and daughters can be different, which can lead to differences in dietary intakes and food choices between them.

Results of our meta‐analysis revealed that familial similarity in the intake of macronutrients as a percentage of energy is higher compared to the conditions in which the intake of macronutrients for individuals has been determined as grams per day; according to the results of meta‐regression, this can be considered as a source of heterogeneity in the results of the studies. The above‐mentioned finding supports the claim that the similarity of genetic characteristics in parent–child can cause them to act similarly in terms of allocating a specific share of daily energy intake for different macronutrients (carbohydrates, protein, and fats). Therefore, although the intake of macronutrients by parents and children as g/day may not be very close to each other, it seems that the genes or single nucleotide polymorphisms (SNPs) involved in determining the percentage of macronutrients from total energy in the form of a dietary pattern for parents and children are similar, and therefore, this has caused that the correlations for the intake of macronutrients as a percentage of energy has been observed stronger than their intakes as g/day among parent–child pairs.

Our findings based on a meta‐regression showed that the resemblance of dietary intake between older children and their parents was lower than their younger counterparts; some previous investigations have suggested the considerable effects of factors outside the home as reasons for these results (Beydoun & Wang, 2009; Hosseini‐Esfahani et al., 2022). As children grow older, they spend more time outside the home and are more likely to be influenced by their peers. Thus, during adolescence and youth, offspring show more autonomy in their food choices and may behave differently than their parents in dietary intake (Beydoun & Wang, 2009). One of the sources of heterogeneity in the results of the present meta‐analysis was the issue of coexistence; we showed that the family similarities in dietary intakes among parent–child not living together versus those living together were different. The overall lower dietary correlations reported in the children not living with their parents revealed that family resemblance and heritability in dietary intakes are not considerable in offspring living apart from their parents and can weaken with time. The influence of other people, such as peers, and also higher autonomy in food choices are factors that cause children who live apart from their parents to make possibly more changes in their food choices than those who live at home with parents (Hosseini‐Esfahani et al., 2022; Lahmann et al., 2017; Zuercher et al., 2011).

In the current meta‐analysis, the year of the study was a source of heterogeneity in the extracted findings. We showed that studies performed before 2000 have stronger family similarities in macronutrient intake among parent–child pairs than studies conducted after 2000; these results revealed that the diversity in food intakes in individuals in recent years (after 2000) was higher in compared to several decades ago (before 2000) because, in times before 2000, individuals mostly adhered to a traditional dietary pattern that was mainly consumed in the family environment, this is even though in recent years, with the occurrence of nutrition transition in various societies (Popkin et al., 2012), individuals' dietary patterns have undergone extensive alteration and they shifted from the traditional and steady dietary pattern to the consumption of fast foods, which are usually consumed outside the home environment (Oexle et al., 2015; Popkin et al., 2012). Also, the extensive changes and developments in the food industry especially in Western countries, and creating various changes in cooking, and preparation methods of foods caused family members, including parents and child may have different types of food in their daily meal plan, some of which foods may be consumed outside the family environment, such as fast‐food restaurants, coffee shops, workplaces, etc. (Oexle et al., 2015; Popkin et al., 2012; Tansey & Worsley, 2014).

Based on our findings, the difference in the dietary intake assessment approach (FFQ vs. food record or 24‐hour recall) was another source of heterogeneity in the findings of studies. We indicated that in the studies that used FFQ to obtain nutritional information, the family similarities observed for different parent–child pairs were weaker than in studies that used the 24‐hour recall or food record for the assessment of dietary intakes. These results can be justified and accepted; because it has been previously demonstrated that using FFQs for the collection of food intake data has major limitations in comparison to other dietary assessment approaches, such as diet diaries or dietary recalls (Olafsdottir et al., 2006). Indeed, FFQs are not optimal nutritional assessment instruments to determine actual dietary intakes. FFQ may not reflect lifetime dietary habits and only provides a general estimation of dietary intakes, while to assess the absolute intake of energy and macronutrients, using a 24‐h recall (for several days) or food diaries may be much more useful and practical (Olafsdottir et al., 2006; Teucher et al., 2007). Thus, FFQ that had been used to collect dietary intake data in several familial studies was a source of heterogeneity (in the form of a negative factor) that has caused a decrease in the reported values of correlation coefficients for energy and macronutrient intakes among parent–child pairs.

The most important application of the results of this meta‐analysis for public health can be that the weak‐to‐moderate similarities in dietary intakes in parent–child pairs show that modification in parents' food habits will have a weak to moderate alteration in children's dietary behaviors; these effects are expected to be seen more in younger children (i.e. in a child less than 10 years old). It is also possible that making dietary interventions in mothers will have a greater impact on improving the food choices of children, especially girls.

Several strengths of this study deserve mention. This meta‐analysis is the first to review and summarize the evidence from all previous studies that examined familial similarity in dietary intakes among different parent–child pairs. We also included in this meta‐analysis as much as possible any study that had examined familial similarity for each macronutrient or energy intake in each parent–child pair, so that we could conduct a comprehensive review of study findings for all macronutrients and energy intake. Furthermore, it should be noted the included studies in this meta‐analysis have been conducted in different societies which have people with different demographic, socioeconomic, and nutritional characteristics, therefore, the results of the present meta‐analysis on the possible family similarities of the intakes of energy and macronutrients may be possibly generalized to different populations. Nevertheless, it is necessary to mention some limitations. Most of the studies on family similarity in dietary intakes among parent–offspring have been conducted in Western countries or developed societies, and studies in developing countries are limited. Considering that lifestyles, nutritional behaviors, and as well as family relationships can be different in these societies, therefore, it is necessary to obtain an accurate summary of the degree of family similarity in dietary intakes, more studies should be performed in developing countries. Also, the meta‐regression analysis in this study showed the possible heterogeneity in the findings of eligible studies conducted on the similarities in intakes of energy and macronutrients among various parent–child pairs. The year of publication of the paper, the dietary assessment approach, the coexistence of parent–child, the child gender, and child age were the sources of heterogeneity in the current study; this heterogeneity in the selected studies may lead to the problem of making a single and accurate conclusion based on extracted findings. Furthermore, Due to the lack of sufficient data in the studies included in the present meta‐analysis, we were unable to determine whether children >18 years old living in the same household as the parent or apart from them, how much it affects the parent–child correlation in energy and macronutrient intakes.

## 5 CONCLUSIONS

The present meta‐analysis suggested that family resemblance of dietary intakes in various parent–offspring pairs can be different based on family pairs, nutrients, and studies. In general, family similarity for dietary intakes, including energy, carbohydrate, fat, and protein was weak in all different parent–child pairs. The strongest dietary intake associations were observed in mother–daughter pairs in which the highest correlation was for carbohydrate and protein intake, respectively. The weakest correlations for dietary intakes were mainly shown in mother–son or father–son pairs. Findings of the current investigation reported that although dietary intakes of people may somewhat be influenced by genetic affinities between the parents and child, the noticeable influence of environmental factors should not be ignored. The weak family similarities of energy and macronutrient intake among various parent–child pairs indicate the effect of environmental factors on individuals' dietary choices, such as the effect of peers, school and classmates, workplace, etc.

## AUTHOR CONTRIBUTIONS

**Farshad Teymoori:** Conceptualization (equal); methodology (equal); writing – original draft (equal); writing – review and editing (equal). **Mostafa Norouzzadeh:** Conceptualization (equal); writing – original draft (equal). **Hossein Farhadnejad:** Methodology (equal); writing – original draft (equal). **Mitra Kazemi Jahromi:** Methodology (equal); writing – original draft (equal). **Hamid Ahmadirad:** Data curation (equal); writing – original draft (equal). **Niloufar Saber:** Data curation (equal); writing – original draft (equal). **Mahdi Akbarzadeh:** Methodology (equal); writing – original draft (equal). **Maryam Zarkesh:** Methodology (equal); writing – original draft (equal). **Maryam S. Daneshpour:** Supervision (equal); writing – review and editing (equal). **Parvin Mirmiran:** Supervision (equal). **mohammadreza vafa:** Conceptualization (equal); investigation (equal); methodology (equal); supervision (equal).

## FUNDING INFORMATION

This study was supported by the Research Institute of Endocrine Sciences, Shahid Beheshti University Medical Sciences, Tehran, Iran.

## CONFLICT OF INTEREST STATEMENT

The authors declare that they have no competing interests.

## ETHICS STATEMENT

The study protocol was approved by the Ethics Committee of the Research Institute for Endocrine Sciences at the Shahid Beheshti University of Medical Sciences.

## CONSENT FOR PUBLICATION

Not applicable.

## Supporting information


Figure S1.

Table S1.

## Data Availability

The data used and/ or analyzed in the present study are available from the corresponding author on reasonable request.

## References

[fsn33957-bib-0001] Adelekan, D. A. , & Adeodu, O. O. (1997). Interrelationship in nutrient intake of Nigerian mothers and their children: Nutritional and health implications. African Journal of Medicine and Medical Sciences, 26(1–2), 63–65. 10895233

[fsn33957-bib-0002] Beydoun, M. A. , & Wang, Y. (2009). Parent‐child dietary intake resemblance in the United States: Evidence from a large representative survey. Social Science & Medicine, 68(12), 2137–2144. 10.1016/j.socscimed.2009.03.029 19375837 PMC2730650

[fsn33957-bib-0003] Bogl, L. H. , Mehlig, K. , Ahrens, W. , Gwozdz, W. , de Henauw, S. , Molnár, D. , Moreno, L. , Pigeot, I. , Russo, P. , Solea, A. , Veidebaum, T. , Kaprio, J. , Lissner, L. , Hebestreit, A. , & IDEFICS and I. Family Consortia . (2020). Like me, like you – relative importance of peers and siblings on children's fast food consumption and screen time but not sports club participation depends on age. International Journal of Behavioral Nutrition and Physical Activity, 17(1), 50. 10.1186/s12966-020-00953-4 32295621 PMC7160987

[fsn33957-bib-0004] Bogl, L. H. , Silventoinen, K. , Hebestreit, A. , Intemann, T. , Williams, G. , Michels, N. , Molnár, D. , Page, A. S. , Pala, V. , Papoutsou, S. , Pigeot, I. , Reisch, L. A. , Russo, P. , Veidebaum, T. , Moreno, L. A. , Lissner, L. , & Kaprio, J. (2017). Familial resemblance in dietary intakes of children, adolescents, and parents: Does dietary quality play a role? Nutrients, 9(8), 892. 10.3390/nu9080892 28817074 PMC5579685

[fsn33957-bib-0005] Boynton‐Jarrett, R. , Thomas, T. N. , Peterson, K. E. , Wiecha, J. , Sobol, A. M. , & Gortmaker, S. L. (2003). Impact of television viewing patterns on fruit and vegetable consumption among adolescents. Pediatrics, 112(6 Pt 1), 1321–1326. 10.1542/peds.112.6.1321 14654604

[fsn33957-bib-0006] Donner, A. , & Rosner, B. (1980). On inferences concerning a common correlation coefficient. Journal of the Royal Statistical Society: Series C: Applied Statistics, 29(1), 69–76.

[fsn33957-bib-0007] Feunekes, G. I. , de Graaf, C. , Meyboom, S. , & van Staveren, W. A. (1998). Food choice and fat intake of adolescents and adults: Associations of intakes within social networks. Preventive Medicine, 27(5 Pt 1), 645–656. 10.1006/pmed.1998.0341 9808794

[fsn33957-bib-0008] Feunekes, G. I. , Stafleu, A. , de Graaf, C. , & van Staveren, W. A. (1997). Family resemblance in fat intake in The Netherlands. European Journal of Clinical Nutrition, 51(12), 793–799. 10.1038/sj.ejcn.1600494 9426352

[fsn33957-bib-0009] French, S. A. , Story, M. , & Jeffery, R. W. (2001). Environmental influences on eating and physical activity. Annual Review of Public Health, 22, 309–335. 10.1146/annurev.publhealth.22.1.309 11274524

[fsn33957-bib-0010] Hosseini‐Esfahani, F. , Zahedi, A. S. , Akbarzadeh, M. , Seyedhamzehzadeh, A. , Daneshpour, M. S. , Mirmiran, P. , & Azizi, F. (2022). The resemblance of dietary intakes in three generations of parent‐offspring pairs: Tehran lipid and glucose study. Appetite, 169, 105794. 10.1016/j.appet.2021.105794 34742772

[fsn33957-bib-0011] Lahmann, P. H. , Williams, G. M. , Najman, J. M. , & Mamun, A. A. (2017). Mother‐adult offspring resemblance in dietary intake: A community‐based cohort study in Australia. The American Journal of Clinical Nutrition, 105(1), 185–193. 10.3945/ajcn.116.137539 27852616

[fsn33957-bib-0012] Laskarzewski, P. , Morrison, J. A. , Khoury, P. , Kelly, K. , Glatfelter, L. , Larsen, R. , & Glueck, C. J. (1980). Parent‐child nutrient intake interrelationships in school children ages 6 to 19: The Princeton School District Study. The American Journal of Clinical Nutrition, 33(11), 2350–2355. 10.1093/ajcn/33.11.2350 7435415

[fsn33957-bib-0013] Lee, H. A. , & Park, H. (2015). Correlations between poor micronutrition in family members and potential risk factors for poor diet in children and adolescents using Korean National Health and nutrition examination survey data. Nutrients, 7(8), 6346–6361. 10.3390/nu7085286 26247964 PMC4555125

[fsn33957-bib-0014] Longbottom, P. , Wrieden, W. , & Pine, C. (2002). Is there a relationship between the food intakes of Scottish 5½−8½‐year‐olds and those of their mothers? Journal of Human Nutrition and Dietetics, 15(4), 271–279. 12153500 10.1046/j.1365-277x.2002.00374.x

[fsn33957-bib-0015] Mitchell, B. D. , Rainwater, D. L. , Hsueh, W. C. , Kennedy, A. J. , Stern, M. P. , & Maccluer, J. W. (2003). Familial aggregation of nutrient intake and physical activity: Results from the San Antonio Family Heart Study. Annals of Epidemiology, 13(2), 128–135. 10.1016/s1047-2797(02)00255-7 12559672

[fsn33957-bib-0016] Nicklas, T. A. , Morales, M. , Linares, A. , Yang, S. J. , Baranowski, T. , De Moor, C. , & Berenson, G. (2004). Children's meal patterns have changed over a 21‐year period: The Bogalusa Heart Study. Journal of the American Dietetic Association, 104(5), 753–761. 10.1016/j.jada.2004.02.030 15127060

[fsn33957-bib-0017] Oexle, N. , Barnes, T. L. , Blake, C. E. , Bell, B. A. , & Liese, A. D. (2015). Neighborhood fast food availability and fast food consumption. Appetite, 92, 227–232. 10.1016/j.appet.2015.05.030 26025087 PMC4500533

[fsn33957-bib-0018] Olafsdottir, A. S. , Thorsdottir, I. , Gunnarsdottir, I. , Thorgeirsdottir, H. , & Steingrimsdottir, L. (2006). Comparison of women's diet assessed by FFQs and 24‐hour recalls with and without underreporters: Associations with biomarkers. Annals of Nutrition & Metabolism, 50(5), 450–460. 10.1159/000094781 16877864

[fsn33957-bib-0019] Oliveria, S. A. , Ellison, R. C. , Moore, L. L. , Gillman, M. W. , Garrahie, E. J. , & Singer, M. R. (1992). Parent‐child relationships in nutrient intake: The Framingham Children's Study. The American Journal of Clinical Nutrition, 56(3), 593–598. 10.1093/ajcn/56.3.593 1503074

[fsn33957-bib-0020] Park, H. S. , Yim, K. S. , & Cho, S. I. (2004). Gender differences in familial aggregation of obesity‐related phenotypes and dietary intake patterns in Korean families. Annals of Epidemiology, 14(7), 486–491. 10.1016/j.annepidem.2003.10.007 15301785

[fsn33957-bib-0021] Pérusse, L. , Tremblay, A. , Leblanc, C. , Cloninger, C. R. , Reich, T. , Rice, J. , & Bouchard, C. (1988). Familial resemblance in energy intake: Contribution of genetic and environmental factors. The American Journal of Clinical Nutrition, 47(4), 629–635. 10.1093/ajcn/47.4.629 3354487

[fsn33957-bib-0022] Popkin, B. M. (2006). Global nutrition dynamics: The world is shifting rapidly toward a diet linked with noncommunicable diseases. The American Journal of Clinical Nutrition, 84(2), 289–298. 10.1093/ajcn/84.1.289 16895874

[fsn33957-bib-0023] Popkin, B. M. , Adair, L. S. , & Ng, S. W. (2012). Global nutrition transition and the pandemic of obesity in developing countries. Nutrition Reviews, 70(1), 3–21. 10.1111/j.1753-4887.2011.00456.x 22221213 PMC3257829

[fsn33957-bib-0024] Rossow, I. , & Rise, J. (1994). Concordance of parental and adolescent health behaviors. Social Science & Medicine, 38(9), 1299–1305. 10.1016/0277-9536(94)90193-7 8016693

[fsn33957-bib-0025] Salvy, S. J. , Romero, N. , Paluch, R. , & Epstein, L. H. (2007). Peer influence on pre‐adolescent girls' snack intake: Effects of weight status. Appetite, 49(1), 177–182. 10.1016/j.appet.2007.01.011 17363109

[fsn33957-bib-0026] Satia, J. A. , Kristal, A. R. , Curry, S. , & Trudeau, E. (2001). Motivations for healthful dietary change. Public Health Nutrition, 4(5), 953–959. 10.1079/phn2001157 11784408

[fsn33957-bib-0027] Savage, J. S. , Fisher, J. O. , & Birch, L. L. (2007). Parental influence on eating behavior: Conception to adolescence. The Journal of Law, Medicine & Ethics, 35(1), 22–34. 10.1111/j.1748-720X.2007.00111.x PMC2531152 17341215

[fsn33957-bib-0028] Shariff, Z. M. , & Yasin, Z. M. (2005). Correlates of children's eating attitude test scores among primary school children. Perceptual and Motor Skills, 100(2), 463–472. 10.2466/pms.100.2.463-472 15974357

[fsn33957-bib-0029] Shrivastava, A. , Murrin, C. , Sweeney, M. R. , Heavey, P. , & Kelleher, C. C. (2013). Familial intergenerational and maternal aggregation patterns in nutrient intakes in the Lifeways Cross‐Generation Cohort Study. Public Health Nutrition, 16(8), 1476–1486. 10.1017/s1368980012003667 22883601 PMC10271879

[fsn33957-bib-0030] Stafleu, A. , Van Staveren, W. A. , de Graaf, C. , Burema, J. , & Hautvast, J. G. (1994). Family resemblance in energy, fat, and cholesterol intake: A study among three generations of women. Preventive Medicine, 23(4), 474–480. 10.1006/pmed.1994.1065 7971875

[fsn33957-bib-0031] Stanton, C. A. , Fries, E. A. , & Danish, S. J. (2003). Racial and gender differences in the diets of rural youth and their mothers. American Journal of Health Behavior, 27(4), 336–347. 12882427 10.5993/ajhb.27.4.5

[fsn33957-bib-0032] Tansey, G. , & Worsley, A. (2014). The food system. Routledge.

[fsn33957-bib-0033] Teucher, B. , Skinner, J. , Skidmore, P. M. , Cassidy, A. , Fairweather‐Tait, S. J. , Hooper, L. , Roe, M. A. , Foxall, R. , Oyston, S. L. , Cherkas, L. F. , Perks, U. C. , Spector, T. D. , & MacGregor, A. J. (2007). Dietary patterns and heritability of food choice in a UK female twin cohort. Twin Research and Human Genetics, 10(5), 734–748. 10.1375/twin.10.5.734 17903115

[fsn33957-bib-0034] Vauthier, J. M. , Lluch, A. , Lecomte, E. , Artur, Y. , & Herbeth, B. (1996). Family resemblance in energy and macronutrient intakes: The Stanislas Family Study. International Journal of Epidemiology, 25(5), 1030–1037. 10.1093/ije/25.5.1030 8921491

[fsn33957-bib-0035] Wang, Y. , Beydoun, M. A. , Li, J. , Liu, Y. , & Moreno, L. A. (2011). Do children and their parents eat a similar diet? Resemblance in child and parental dietary intake: Systematic review and meta‐analysis. Journal of Epidemiology and Community Health, 65(2), 177–189. 10.1136/jech.2009.095901 21051779 PMC3010265

[fsn33957-bib-0036] Wang, Y. , Li, J. , & Caballero, B. (2009). Resemblance in dietary intakes between urban low‐income African‐American adolescents and their mothers: The healthy eating and active lifestyles from school to home for kids study. Journal of the American Dietetic Association, 109(1), 52–63. 10.1016/j.jada.2008.10.009 19103323 PMC2643250

[fsn33957-bib-0037] Wroten, K. C. , O'Neil, C. E. , Stuff, J. E. , Liu, Y. , & Nicklas, T. A. (2012). Resemblance of dietary intakes of snacks, sweets, fruit, and vegetables among mother‐child dyads from low income families. Appetite, 59(2), 316–323. 10.1016/j.appet.2012.05.014 22634195

[fsn33957-bib-0038] Zuercher, J. L. , Wagstaff, D. A. , & Kranz, S. (2011). Associations of food group and nutrient intake, diet quality, and meal sizes between adults and children in the same household: A cross‐sectional analysis of U.S. households. Nutrition Journal, 10(1), 131. 10.1186/1475-2891-10-131 22123043 PMC3281797

