# Supplementary material for: Parent–child correlation in energy and macronutrient intakes: A meta‐analysis and systematic review
Source: Food Sci Nutr. 2024 Jan 17;12(4):2279–93. doi: 10.1002/fsn3.3957 (PMC11016429; doi:10.1002/fsn3.3957)
Supplement: Supplementary file 2 — Table S1. [file FSN3-12-2279-s001.docx]

**Supplementary Table 1.** Quality Assessment using Newcastle-Ottawa Scale for Cross-sectional studies^1^

|  | **Selection** | | | **Comparability** | **Outcome** | | Study  score |
| --- | --- | --- | --- | --- | --- | --- | --- |
| Study | Representativeness of the exposed sample | Selection of the non-Exposed sample | Ascertainment of exposure | Comparability of Outcome groups on the  Basis of design or analysis | Assessment of outcome | The statistical test is appropriate |  |
| Park et al, 2004 | * | ** | ** | * | ** | * | 9/10 |
| Lee et al, 2015 | * | ** | ** | * | ** | * | 9/10 |
| Mitchell et al, 2003 | - | ** | ** | * | ** | * | 8/10 |
| Perusse et al, 1988 | * | ** | ** | * | ** | * | 9/10 |
| Vauthier et al, 1996 | * | ** | ** | * | ** | * | 9/10 |
| Bogl et al, 2017 | * | ** | ** | * | ** | * | 9/10 |
| Feunekes et al, 1997 | * | ** | * | * | ** | * | 8/10 |
| Feunekes et al, 1998 | * | ** | ** | * | ** | * | 9/10 |
| Rossow et al, 1994 | * | ** | * | * | ** | * | 8/10 |
| Stafleu et al, 1994 | * | ** | ** | * | ** | * | 9/10 |
| Adelekan et al, 1997 | * | ** | ** | * | ** | * | 9/10 |
| Oliveria et al, 1992 | - | ** | * | * | ** | * | 7/10 |
| Longbottom et al, 2002 | * | ** | ** | * | ** | * | 9/10 |
| Stanton et al, 2003 | * | ** | * | * | ** | * | 8/10 |
| Wang et al, 2009 | * | ** | ** | * | ** | * | 9/10 |
| Beydoun et al, 2009 | * | ** | ** | * | ** | * | 9/10 |
| Laskarzewski et al, 1980 | * | ** | * | * | ** | * | 8/10 |
| Wroten et al, 2012 | * | ** | ** | * | ** | * | 9/10 |
| Lahmann et al, 2017 | * | ** | ** | * | ** | * | 9/10 |
| Shrivastava et al, 2013 | * | ** | * | * | ** | * | 8/10 |
| Hosseini-Esfahani et al, 2022 | * | ** | ** | * | ** | * | 9/10 |

*^1^We used a modified NOS scale for cross-sectional studies. Note: A score of 7 or higher was considered a good study.*
